# Statistical Inference in Database Exploitation: Sample Size and Confounding Factors

**DOI:** 10.31083/RN52643

**Published:** 2026-06-30

**Authors:** Carmen Carazo-Díaz, Luis Prieto-Valiente

**Affiliations:** ^1^Faculty of Medicine, Catholic University of Murcia (UCAM), E-30107 Murcia, Spain; ^2^Catholic University of Murcia (UCAM) and Scientific Society of Biomedical Research (SCIB), 07010 Palma de Mallorca, Spain

**Keywords:** database, medical research, sample size, multivariate analysis, confounding variables

## Abstract

Database (DB) exploitation is an essential tool in medical and epidemiological research, enabling the extraction of hidden insights from large volumes of information through statistical analysis. This work provides a methodological reflection on two critical aspects of such studies, namely sample size and confounder detection via multivariate analysis. When working with databases, researchers must perform statistical inference based exclusively on the available data and therefore often ask whether the fixed sample size is sufficient to detect relationships of interest. In most cases, no threshold value separates valid sizes from invalid ones, as statistical power increases gradually and depends on multiple parameters, with no fixed cutoff points. Even when the results are inconclusive (high *p*-values), possibly because of limited sample size, their publication is essential to feed future meta-analyses that may provide more solid conclusions. DB analysis requires discriminating between general and circumstantial associations. Although the relationships revealed by univariate analysis maintain their descriptive value, the accuracy of their interpretation can be increased by identifying possible confounding factors.

## 1. Introduction

Recent technological developments have enabled the orderly collection and archiving of large amounts of information in all areas of science (including medicine and sociology) and life in general. These databases (DBs) contain useful information that is not visible at first glance but can be revealed by descriptive and inferential statistical research. Access to data in this study variant has specific peculiarities, such as selection bias, information bias, and data quality, which merit careful attention and detailed comments that may be addressed in subsequent articles. Herein, we focus on two areas where researchers without statistical training most frequently need specific advice, namely sample size and multivariate analysis–based confounder detection.

## 2. Sample Size

When working with DBs, researchers can only perform inference using the available individuals, i.e., the sample size is fixed. The corresponding *p*-values indicate the degree to which the data contradict the null hypothesis proposed at each moment and support the hypothesis that the effect found in the sample also occurs in the population. If the *p*-value is relatively large, i.e., if the significance of the result is relatively low, the sample size cannot be increased, although prospective and retrospective studies can be subsequently conducted to test the same hypothesis.

Suppose that according to the examined DB, severe migraine affects 10% of sedentary people and 3% of athletes, the advantage in favor of sedentary people being 7 percentage points. Let us examine the effects of DB size on the results of statistical inference. For 500 people in each group, *p* < 0.00001, the 95% confidence interval (CI) for the difference in population proportions is (4% and 10%), and the effect size (Cohen’s *h*) is 0.29 with a 95% CI of [0.163, 0.413], i.e., the result is very conclusive. For 50 people in each group, *p* = 0.16, the 95% CI for the difference in population proportions is (–3% and 16%), and the 95% CI for the effect (Cohen’s *h*) is [–0.108, 0.684], i.e., the result is not conclusive, and more information is required. However, the latter result deserves publication because it could be very useful when evaluated alongside other information on the subject—for example, helping a meta-analysis yield more solid conclusions.

One often wonders whether a given sample size is sufficient to detect relationships of interest. Contrary to a fairly widespread error among researchers, there is no threshold value that separates valid from invalid sample sizes [1, 2, 3, 4]. The basic logic of statistical inference indicates that the ability to detect population relationships—that is, the statistical power of the study—increases progressively with sample size. This gradual transition has no cutoff points or boundary values that separate sufficient sizes from insufficient ones. For example, it is obvious that a power of 12% is very low and that of 97% is very high. However, it is equally obvious that there is no tipping point separating low- and high-power zones. The related decision-making relies on basic logic that concerns both this parameter and many others in science and life in general. Moreover, the statistical power of an investigation is NOT a fixed value specific to each study but depends on sample size and other parameters, which, in turn, are not univocally established [5, 6].

Therefore, in most cases, it is incorrect to say that the DB size is (in)adequate to provide sufficient statistical power. The related misconception ecosystem is deeply rooted in the research and should be addressed through systematic educational efforts focused on the following basic concepts.

(A) No specific value separating sufficient and insufficient power exists.

(B) The power of a contrast depends on the variability of the variability for a quantitative variable and the relative frequency of those cured with placebo for a qualitative variable, data that are not precisely known.

(C) The power of a contrast depends on the alpha value that is agreed upon as significant; however, this value can broadly vary.

(D) The power of a contrast depends on the magnitude of the real effect (that in the population), which is not known by definition, as elucidating this magnitude is the aim of the study.

(E) Every data analysis involves several variables, each with its own estimates of variability, agreed alpha value, and real effect taken as a reference.

However, indicative estimates can be made to determine what levels (approximately) of power correspond to what levels (approximately) of the alpha value, real effect, and variability. For example, if a certain dichotomous characteristic in a population is found in 60% of women and 50% of men, for a DB with *N* = 1600, the power to detect this difference with a *p*-value of <0.05 is ~97.3%. For population percentages of 10% and 20%, respectively, the power is ~99.9%. For a quantitative variable, the power to detect (*p* < 0.05, two-tailed) a real mean difference equivalent to 0.2 deviations is 97.5%.

Finally, although sample size determines the ability to reach statistical significance, the clinical utility of a study transcends the volume of data. The relevance of the findings depends on the solidity of the research question, which defines the relevance of the analysis; the frequency of the results, essential to guarantee power in rare events; data variability; and the required precision, which establishes the margin of rigor necessary for a valid clinical interpretation.

## 3. Confounding and Intermediate Factors

The interpretation and practical impact of confounding factors depend on the type of sampling and the objective of each study phase. In general, general associations should be distinguished from circumstantial associations in the current sample [7]. Furthermore, the conclusion reached when exploiting a DB may be different from that reached when two or more independent samples are taken, as explained below.

Suppose that according to our DB, severe migraine affects 10% of men and 27.5% of women, with the exploration of anemia as a possible confounding factor revealing the following results (Table 1).

**Table 1.** **Number and proportion of migraine cases according to sex and presence or absence of anemia**.

	Men			Women		
	Total	Migraine	Migraine (%)	Total	Migraine	Migraine (%)
All	10,000	1000	10.0%	10,000	2750	27.5%
No anemia	8000	400	5.0%	1000	50	5.0%
Anemia	2000	600	30.0%	9000	2700	30.0%

(A) In people with anemia, migraine affects men and women equally (30% in each sex).

(B) In people without anemia, migraine also affects men and women equally (5% in each sex).

(C) In the raw data, migraine is more frequent in women (27.5%) than in men (10%).

In this case, anemia is a clear confounding factor, and the correct thing to do is ignore the raw effect and conclude that sex and suffering from migraine are unrelated to each other. Although this is true, the following nuances should be considered.

If the higher proportion of anemia in women in the sample (90% compared with 20% in men) reflects that at the population level, we conclude that in the population, migraine is more frequent in women because anemia is more frequent in them, i.e., anemia is a part of the mechanism that makes migraine more frequent in women. The fact that the greater presence of anemia in women (90% vs. 20%) is the cause of the greater presence of migraine in them (27.5% vs. 10%) does not change this fact and its epidemiological importance. Similarly, any other circumstantial association between two variables does not have less relevance if it may be mediated by intermediate factors.

Therefore, if the initial analysis of the DB aims to reveal empirical relationships between variables, the associations revealed by univariate analysis are not definitively invalidated by possible confounders [8, 9]. The clinical and epidemiological importance of each relationship is not compromised by the detection of intermediate factors. In fact, the identification of confounders through multivariate analysis can help better understand the action mechanisms involved in each univariate relationship.

In a scenario where the data in Table 1 are not found in a DB but refer to four random samples (8000 men without anemia, 2000 men with anemia, 1000 women without anemia, and 9000 women with anemia), the situation is very different. In this case, we conclude that anemia appears more frequently in women because we have chosen to take samples of these sizes, with the 90% of anemia in women and 20% in men not reflecting any population value. We find that in our samples, migraines are equally frequent in both sexes, and there is no indication that the corresponding frequencies are different in the population. In this case, anemia is a confounding factor. The values of 10% and 27.5% in the first row, which suggest a general association between sex and migraine, are numerical artifacts that do not provide useful information because they do not represent population values.

The above reasoning is not mathematical but logical and can be difficult to follow for professionals insufficiently familiar with data analysis. Even statisticians need to make efforts to capture the essence of these relationships and draw correct conclusions, depending on the type of sampling and study objective. Most doctors and other health professionals need to make even greater and not always successful efforts. Hence, information of this type is most efficiently analyzed when doctors and data analysts work together.

Consider another very intuitive example of circumstantial association: Among primiparous mothers, the percentage of newborns with hypoxia problems due to prolonged labor is much higher in a certain private hospital A that serves high-economic-level women than in a public hospital B that serves low-economic-level women. This information is useful for making specialized care plans for hypoxia-affected newborns. Multivariate analysis reveals that age is a decisive confounding factor, as primiparous women at A are much older on average than those at B. This indicates that high economic level is not the cause of the higher incidence of hypoxia; however, the proportion of newborns with hypoxia is still higher in A, and knowing this helps provide the necessary resources.

The following two statements are true and compatible, although this is not obvious at first glance.

(A) The proportion of newborns with hypoxia is much higher in hospital A than in hospital B.

(B) Giving birth in hospital A does NOT entail more risk of having a newborn with hypoxia than giving birth in hospital B. Each mother’s risk depends on her age, not the hospital.

The data suggest that to minimize the risk of a birth with hypoxia, the most effective strategy is not choosing hospital B but advancing the maternal age.

## 4. Conclusions

When exploring DBs, researchers do not determine the sample size but are rather tasked with performing statistical inference on the set of available individuals. Contrary to a common belief among professionals with little training in statistical analysis, no numerical threshold exists that separates valid from invalid sample sizes. Conceptually, there is no critical cutoff value, and questioning whether the DB size is sufficient to detect relationships of interest is therefore inappropriate.

The interpretation of confounding factors and their practical impact directly depends on the sampling nature and objectives of each study phase. General associations should be distinguished from circumstantial ones in the current sample. The conclusions drawn for a single DB may differ from those drawn when two or more independent samples are taken. If the initial DB analysis aims to identify empirical relationships between variables, the associations detected byunivariate analysis retain their descriptive value, although multivariate analysis can enrich the study by helping explain the involved action mechanisms.

## Data Availability

The data contained in the manuscript are fictitious and used for educational purposes.
