# Supplementary material for: Statistical Inference in Database Exploitation: Sample Size and Confounding Factors
Source: Rev Neurol. 2026 Jun 30;81(6):52643. doi: 10.31083/RN52643 (PMC13339777; doi:10.31083/RN52643)
Supplement: Translation

# Translation: Inferencia Estadística al Explotar una Base de Datos. Tamaño de la Muestra y Factores Confusores

**Published:** 2026-06-30

**Authors:** Carmen Carazo-Díaz, Luis Prieto-Valiente

**Affiliations:** ^3^Facultad de Medicina, Universidad Católica de Murcia (UCAM), E-30107 Murcia, España; ^4^Sociedad Científica de Investigación Biomédica (SCIB), 07010 Palma de Mallorca, España

**Keywords:** bases de datos, investigación médica, tamaño de la muestra, análisis multivariante, variables de confusión

## Abstract

La explotación de bases de datos (BD) se ha consolidado como una herramienta esencial en la investigación médica y epidemiológica, permitiendo extraer “tesoros escondidos” de grandes volúmenes de información mediante el análisis estadístico. El objetivo de este trabajo es hacer una reflexión metodológica sobre dos aspectos críticos y especialmente relevantes en este tipo de estudios: El tamaño de la muestra y el Análisis multivariado para detectar confusores. En esta modalidad de estudio, el investigador no determina el tamaño de la muestra, sino que debe realizar la inferencia estadística basándose exclusivamente en los datos disponibles. No siendo modificable el tamaño de la muestra, teóricamente cabría preguntarse si el que tiene la BD es suficiente para detectar relaciones de interés. La respuesta es que, en la mayoría de los casos no hay un número de tamaño de muestra que separe los tamaños “válidos” de los que no lo son; la potencia estadística crece de forma gradual y depende de múltiples parámetros, sin puntos de corte fijos. Incluso cuando los resultados no son concluyentes (valores *p* elevados), quizá debido a un tamaño muestral limitado, estos conservan su valor científico, ya que su publicación es esencial para alimentar futuros metaanálisis que aporten conclusiones más sólidas. Finalmente, el análisis de estas bases de datos requiere diferenciar las asociaciones generales de las circunstanciales. Si bien las relaciones halladas en los análisis univariados mantienen un valor descriptivo relevante, este análisis se enriquece sustancialmente al identificar posibles factores de confusión para una interpretación precisa de los resultados.

## 1. Introducción

Una de las modalidades cada vez más utilizada en investigación médica y epidemiológica es la explotación de bases de datos, BD. El desarrollo tecnológico creciente de los últimos decenios permite recabar y archivar ordenadamente cantidades ingentes de información en todos los ámbitos de la ciencia y de la vida en general. El ámbito de la medicina y la sociología no es una excepción, sino un exponente máximo de esa realidad. Está fuera de toda duda que esas BD contienen información muy útil no visible a primera vista. Y sacar a la luz esos “tesoros escondidos” es una labor de investigación estadística—tanto descriptiva como inferencial—que aportará notables conocimientos nuevos. El acceso a los datos en esta variante de estudio tiene peculiaridades específicas, como el sesgo de selección, el sesgo de información y la calidad de los datos, que ameritan atención cuidadosa y comentarios detallados que podrán ser abordados en ulteriores artículos. Pero en este breve trabajo nos centramos en dos aspectos especialmente relevantes: El tamaño de la muestra y el Análisis multivariado para detectar confusores, al ser en los que con mayor frecuencia el investigador sin formación estadística puede necesitar asesoramiento específico.

## 2. El tamaño de la Muestra

Respecto al tema del tamaño de muestra debe estar claro que al tratarse de la explotación de una BD el investigador NO es quien elige el tamaño. Lo único que cabe es hacer la inferencia usando los individuos con los que cuenta. Los valores *p* encontrados nos indicarán el grado de evidencia que los datos aportan contra la Hipótesis Nula planteada en cada momento y en favor de que el efecto encontrado en la muestra ocurra también en la población. En los casos en que aparece valor *p* del test relativamente grande (resultado poco significativo) no está en la mano del investigador aumentar el tamaño de la muestra, aunque podría plantearse hacer estudios posteriores, prospectivos y retrospectivos, encaminados a testear esa misma hipótesis.

Como ejemplo supongamos que en la BD que estemos explorando aparece que la jaqueca severa afecta al 10% de las personas sedentarias y al 3% de las deportistas, con una ventaja “a favor” de las sedentarias de 7 puntos porcentuales. Veamos que nos ofrece la Inferencia Estadística según sea el tamaño de la BD. Si había 500 personas en cada grupo, tenemos *p* < 0,00001, intervalo de confianza (IC) 95% para la diferencia de proporciones poblacionales de (4% y 10%) y tamaño del efecto (h de Cohen) = 0.29, con IC 95% del efecto: [0,163, 0,413]. Los datos son muy concluyentes. Pero si había 50 personas en cada grupo, tenemos *p* = 0,16, IC 95% para la diferencia de proporciones poblacionales de (–3% y 16%) e IC 95% del efecto (h de Cohen): [–0,108, 0,684]. El resultado no es concluyente y el tema queda abierto a la espera de que haya más información. Pero sin duda amerita ser publicado porque al pasar a disposición de la comunidad científica podría ser muy útil al evaluarse junto a otra información sobre el tema, por ejemplo, colaborando a que un metaanálisis arroje conclusiones más sólidas.

No siendo modificable el tamaño de la muestra, teóricamente cabría preguntarse si el que tiene nuestra BD es suficiente para detectar relaciones de interés para el investigador. En contra de un error bastante extendido entre los investigadores, la respuesta es que no hay un valor del tamaño de la muestra que separe los tamaños “válidos” de los que no lo son [1, 2, 3, 4]. La lógica básica de la inferencia estadística indica que la capacidad de una muestra para detectar relaciones poblacionales, es decir, la potencia estadística del estudio, aumenta progresivamente a medida que aumenta el tamaño. Es una transición gradual que no tiene puntos de corte ni valores frontera que separen los tamaños “suficientes” de los “insuficientes”. Es obvio que una potencia de, por ejemplo, 12% es muy baja y 97% es muy alta. Pero es igualmente obvio que no hay un punto de inflexión que separe la zona “baja” de la “alta”. Es una cuestión de lógica básica que atañe tanto a este parámetro como a otros muchos de la ciencia y de la vida en general. Hay que tener muy presente, además, que la potencia estadística de una investigación NO es un valor fijo propio de cada estudio, sino una cantidad que depende, además del tamaño de la muestra, de otros parámetros que, a su vez, no están establecidos unívocamente [5, 6].

Sería, por tanto, en la mayoría de los casos, incorrecto decir que el tamaño de una BD es “adecuado” o no para proporcionar una potencia estadística “suficiente”. Es un error muy asentado en el ecosistema de la investigación y es necesario un trabajo docente serio y urgente para deshacer este malentendido, insistiendo en la aclaración de estos conceptos básicos:

(A) No hay un valor concreto de la potencia que separe la potencia “suficiente” de la “insuficiente”.

(B) La potencia de un contraste depende de la variabilidad de la variable implicada (para variable cuantitativa) y de la frecuencia relativa de curados con placebo (para variable cualitativa), datos que no son conocidos con precisión.

(C) La potencia de un contraste depende del valor alfa que se acuerde como “significativo”, y ese acuerdo puede tomar valores muy distintos.

(D) La potencia de un contraste depende de la magnitud del efecto real (en la población), cantidad que—por definición—no se conoce. Si se conociera no habría que hacer ese estudio.

(E) En todo análisis de datos hay implicadas varias variables, cada una de ellas con sus propias estimaciones de la variabilidad, el valor alfa acordado y el efecto real que se toma como referencia.

No obstante, cabe hacer estimaciones orientativas para mostrar qué niveles (aproximadamente) de potencia habría para qué niveles (aproximadamente) de valor alfa y qué valores (aproximadamente) de variabilidad y qué valores (aproximadamente) de efecto real. Por ejemplo, si cierta característica dicotómica concurriera realmente en población en el 60% de las mujeres y el 50% de los varones, con una BD con N = 1600 la potencia para detectar esa diferencia con un valor *p* < 0,05 sería del orden de 97,3%. Y si los porcentajes poblacionales fueran 10% y 20% respectivamente, la potencia sería del orden de 99,9%. Para una variable cuantitativa, la potencia para detectar (*p* < 0,05, bilateral) una diferencia real de medias equivalente a 0,2 desviaciones, es 97,5%.

Por último, puntualizar que, si bien el tamaño de la muestra determina la capacidad para alcanzar significación estadística, la utilidad clínica del estudio trasciende el volumen de datos. La relevancia de los hallazgos a su vez depende de la solidez de la pregunta de investigación, que define la pertinencia del análisis; la frecuencia de los resultados, esencial para garantizar potencia en eventos poco comunes; la variabilidad de los datos y la precisión requerida, que establece el margen de rigor necesario para una interpretación clínica válida.

## 3. Factores de Confusión y Factores Intermedios en las Bases de Datos

La interpretación y repercusión práctica de los factores de confusión varía según sea el tipo de muestreo que nos proporciona los datos y el objetivo de cada fase del estudio. En general hay que distinguir claramente entre asociación general o asociación circunstancial en la muestra actual [7]. Además cuando se trata de explotar una BD la conclusión puede ser distinta de cuando se toman dos o más muestras independientes entre sí. Se explica y se entiende más fácilmente con un ejemplo concreto.

Supongamos que en nuestra BD se encuentra que la jaqueca severa afecta al 10% de los varones y al 27,5% de las mujeres y al explorar la anemia como posible factor de confusión se encuentra que (ver Tabla 1):

**Tabla 1.** **Cantidad y proporción de casos de jaqueca según sexo y presencia o no de anemia**.

	Varones			Mujeres		
	Total	Jaqueca	% Jaqueca	Total	Jaqueca	% Jaqueca
Todos	10.000	1000	10,0%	10.000	2750	27,5%
No anemia	8000	400	5,0%	1000	50	5,0%
Anemia	2000	600	30,0%	9000	2700	30,0%

(A) En las personas con anemia, la jaqueca afecta por igual a varones y a mujeres, 30% en ambos sexos.

(B) En las personas sin anemia, la jaqueca afecta también por igual a varones y mujeres, 5% en ambos sexos.

(C) En los datos brutos la jaqueca es más frecuente en las mujeres (27,5% vs 10%).

Se ve claramente que la anemia es un claro factor confusor y lo correcto es ignorar el efecto bruto y concluir que no se encuentra relación entre el sexo y padecer jaqueca. Pero siendo esto verdad, es necesario tener en cuenta los siguientes matices:

Si la mayor proporción de anemia en las mujeres en la muestra (90% frente a 20% en varones) estima mayor proporción de anemia en las mujeres a nivel poblacional, diremos también en la población la jaqueca es más frecuente en las mujeres debido a que en ellas es más frecuente la anemia y esta conlleva más frecuencia de jaqueca. Es decir, la anemia forma parte del mecanismo que hace más frecuente la jaqueca en las mujeres.

Que la mayor presencia de anemia en mujeres (90% vs 20%) sea la causa de la mayor presencia de jaqueca en las mujeres (27,5% vs 10%) no modifica este hecho, ni su importancia epidemiológica. Del mismo modo, cualquier otra asociación circunstancial que encontremos entre dos variables no tendrá menos relevancia por el hecho de que pueda ser mediada por factores intermedios.

Por ello, si en un primer análisis de la BD buscamos relaciones empíricas entre variables, las asociaciones que aparezcan en el análisis mono-variado no son definitivamente invalidadas por posibles confusores [8, 9]. La importancia clínica y epidemiológica de cada relación permanece aunque se detecten factores intermedios que deban tenerse muy presentes. De hecho el hallazgo de confusores con análisis multivariado, puede ayudar decisivamente a conocer mejor los mecanismos de acción implicados en cada relación univariada.

Pero si los datos de la Tabla 1 no se han encontrado en una BD, sino que se decidió tomar 4 muestras al azar: 8000 varones sin anemia, otra de 2000 varones con anemia, otra de 1000 mujeres sin anemia y otra de 9000 mujeres con anemia, la situación es muy distinta.

En este caso concluimos que la anemia aparece con más frecuencia en mujeres, porque nosotros hemos elegido tomar las muestras de esos tamaños, de modo que el 90% de anemia en mujeres y 20% en varones no estiman ningún valor poblacional. Encontramos que en nuestras muestras las jaquecas son igual de frecuentes en ambos sexos y no hay indicios de que en la población sean distintos. En este caso la anemia es un claro caso de factor confusor. El 10% y el 27,5% de la primera fila numérica, que sugieren una asociación general entre sexo y jaqueca, son artefactos numéricos que no dan información útil pues no estiman valores poblacionales.

El razonamiento seguido no es matemático, sino lógico, pero puede ser un poco dificultoso para el profesional no muy familiarizado con el análisis de datos. Incluso los estadísticos, que conviven a diario con información numérica, necesitan dedicar atención muy cuidadosa para captar lo esencial de estas relaciones y sacar las conclusiones correctas, según sea el tipo de muestreo y el objetivo del estudio. La mayoría de los médicos y otros profesionales de las ciencias de la salud necesitarán un esfuerzo aun mayor y no siempre exitoso. Por eso el análisis más eficiente de este tipo de información se obtiene cuando médicos y analistas de datos trabajan conjuntamente, aunando esfuerzos.

Insistamos en esta idea con otro ejemplo muy intuitivo de asociación circunstancial: Un estudio detectó que entre las madres primíparas el porcentaje de Recién Nacidos (RN) con problemas de hipoxia por parto de larga duración es mucho mayor en cierto hospital privado “A” que atiende a mujeres de alto nivel económico que en el hospital público “B” que atiende a mujeres de bajo nivel económico. Es una información útil para hacer previsiones de atención especializada a los RN con ese problema. La búsqueda de confusores con análisis multivariante reveló que la edad es un factor de confusión decisivo, al tener las primíparas del A mucha más edad media que las del B. Ello nos permite saber que el nivel económico alto no es la causa de la mayor incidencia de hipoxia, pero sigue siendo cierto que en el A la proporción de RN con hipoxia es mayor y saber eso ayuda a proveer de los recursos necesarios.

Las dos afirmaciones que siguen podrían parecer incompatibles, pero ambas son ciertas y compatibles:

(a) La proporción de RN con hipoxia es mucho mayor en el hospital A que en el B.

(b) Parir en el hospital A NO conlleva más riesgo de tener RN con hipoxia que parir en el B. El riesgo de cada madre depende de su edad, no del hospital.

Los datos sugieren que, para minimizar el riesgo de un parto con hipoxia, la estrategia más efectiva para las futuras madres no radica en la elección del hospital B, sino en adelantar la edad gestacional.

## 4. Conclusión

En la explotación de bases de datos preexistentes, el investigador no determina el tamaño de la muestra; su labor se limita a realizar la inferencia estadística sobre el conjunto de individuos disponibles. Existe una creencia errónea—frecuente en profesionales con escasa formación en análisis estadístico—de que existe un umbral numérico que separa los tamaños muestrales “válidos” de los que no lo son. Sin embargo, conceptualmente no existe un valor crítico de corte, por lo que resulta improcedente cuestionar si el volumen de la base de datos es “suficiente” para detectar relaciones de interés.

La interpretación de los factores de confusión y su repercusión práctica dependen directamente de la naturaleza del muestreo y de los objetivos de cada fase del estudio. Es fundamental distinguir entre asociación general o asociación circunstancial en la muestra actual. Cuando se trabaja con una base de datos única, las conclusiones pueden diferir de cuando se toman dos o más muestras independientes entre sí. Si en un primer análisis de la BD el objetivo inicial es identificar relaciones empíricas entre variables, las asociaciones detectadas en el análisis monovariado conservan su valor descriptivo pero el análisis multivariante puede enriquecer decisivamente el estudio colaborando a explicar los mecanismos de acción implicados.

## Data Availability

The data contained in the manuscript are fictitious and used for educational purposes.

## References

[1] Bacchetti P (2010). Current sample size conventions: Flaws, harms, and alternatives. *BMC Medicine*.

[2] Martínez-Sellés M, Prieto L, Herranz I (2005). Frequent mistakes in the statistical inference of biomedical data. *Italian Heart Journal*.

[3] Prieto L, Prieto-Merino D (2003). Errores más frecuentes al elaborar conclusiones en trabajos científicos. *Fisiología*.

[4] Abellán-Huerta J, Prieto-Valiente L (2020). The sample size myth. *Revista Espanola De Cardiologia*.

[5] Hoenig JM, Heisey DM (2001). The abuse of power: the pervasive fallacy of power calculations for data analysis. *The American Statistician*.

[6] Goodman SN, Berlin JA (1994). The use of predicted confidence intervals when planning experiments and the misuse of power when interpreting results. *Annals of Internal Medicine*.

[7] Zurita-Cruz JN, Villasís-Keever MA (2021). Main biases in clinical research. *Revista alergia México*.

[8] Ioannidis JPA (2005). Contradicted and initially stronger effects in highly cited clinical research. *JAMA*.

[9] Argimon JM (2004). Métodos de investigación clínica y epidemiológica.
